# Upregulation of cognitive control networks in older adults’ speech comprehension

**DOI:** 10.3389/fnsys.2013.00116

**Published:** 2013-12-24

**Authors:** Julia Erb, Jonas Obleser

**Affiliations:** Max Planck Research Group “Auditory Cognition”, Max Planck Institute for Human Cognitive and Brain SciencesLeipzig, Germany

**Keywords:** functional MRI, aging, degraded speech, neural adaptation, executive functions, noise-vocoding, cochlear implant simulation, temporal envelope

## Abstract

Speech comprehension abilities decline with age and with age-related hearing loss, but it is unclear how this decline expresses in terms of central neural mechanisms. The current study examined neural speech processing in a group of older adults (aged 56–77, *n* = 16, with varying degrees of sensorineural hearing loss), and compared them to a cohort of young adults (aged 22–31, *n* = 30, self-reported normal hearing). In a functional MRI experiment, listeners heard and repeated back degraded sentences (4-band vocoded, where the temporal envelope of the acoustic signal is preserved, while the spectral information is substantially degraded). Behaviorally, older adults adapted to degraded speech at the same rate as young listeners, although their overall comprehension of degraded speech was lower. Neurally, both older and young adults relied on the left anterior insula for degraded more than clear speech perception. However, anterior insula engagement in older adults was dependent on hearing acuity. Young adults additionally employed the anterior cingulate cortex (ACC). Interestingly, this age group × degradation interaction was driven by a reduced dynamic range in older adults who displayed elevated levels of ACC activity for both degraded and clear speech, consistent with a persistent upregulation in cognitive control irrespective of task difficulty. For correct speech comprehension, older adults relied on the middle frontal gyrus in addition to a core speech comprehension network recruited by younger adults suggestive of a compensatory mechanism. Taken together, the results indicate that older adults increasingly recruit cognitive control networks, even under optimal listening conditions, at the expense of these systems’ dynamic range.

## Introduction

Speech comprehension can become difficult with age and age-related hearing loss, especially when listening conditions are challenging. Normal-hearing young adults have the capacity to rapidly adapt to degraded speech (Davis et al., 2005; Samuel and Kraljic, 2009; Eisner et al., 2010; Erb et al., 2013). Such short-term perceptual adaptation is not well established in older adults, although it bears particular relevance as older adults are frequently affected by hearing loss. For example, patients with hearing-aids or, more drastically, cochlear implants (CI) need to adapt to an altered and often distorted auditory signal delivered by their device.

In a previous short-term adaptation study in a cohort of young adults (Erb et al., 2013), we have shown that degraded speech processing elicits an increased blood oxygenation level-dependent (BOLD) response in an “executive” network (Eckert et al., 2009) comprising the anterior insula and anterior cingulate cortex (ACC). Also, adaptation to degraded speech was shown to be accompanied by hemodynamic down-regulation in a cortico-thalamic-striatal loop (Erb et al., 2013). In the current functional MRI experiment, we compare these results to a group of older adults with varying degrees of hearing loss to test: (1) whether older listeners are able to (behaviorally) adapt to spectrally severely degraded (“noise-vocoded”) speech at a rate comparable to young listeners and (2) how neural processing of degraded speech differs between young and older adults.

There is evidence that rapid perceptual learning is preserved in older adults (Peelle and Wingfield, 2005; Golomb et al., 2007; Gordon-Salant et al., 2010). For example, older adults are able to quickly adapt to an unfamiliar accent (Adank and Janse, 2010) or a foreign accent (Gordon-Salant et al., 2010). Peelle and Wingfield (2005) showed that older adults adapted to temporally degraded (“time-compressed”) and noise-vocoded speech at a similar rate as young adults, when starting accuracy was equated.

However, considerable inter-individual variability has been frequently observed in adaptation to vocoded speech (Shannon et al., 2002; Eisner et al., 2010). Especially in older adults, the degree of hearing loss and cognitive aspects might substantially impact adaptation to degraded speech. Working memory is one cognitive factor that has been implicated in degraded speech comprehension by a number of studies (Pichora-Fuller et al., 1995; Burkholder et al., 2005; Jacquemot and Scott, 2006; Eisner et al., 2010; Piquado et al., 2010; Obleser et al., 2012). For example, Pisoni and Cleary (2003) observed that working memory scores as measured by digit span significantly predicted speech comprehension in pediatric CI users. In older adults, cognitive factors might be even more closely related to degraded speech recognition (Janse and Adank, 2012), because cognitive decline has been shown to co-occur with sensory decline (Lindenberger and Ghisletta, 2009), which in turn leads to degraded auditory conditions. Thus, we expected working memory capacity in older adults to be related to vocoded speech comprehension.

A second factor which heavily affects comprehension is hearing loss. As age-related hearing loss is accompanied by auditory cortex atrophies (Harris et al., 2009; Peelle et al., 2011; Eckert et al., 2012), older adults likely draw on different neural resources for speech comprehension. It is a common observation that older adults recruit additional regions for speech comprehension compared to young adults, although it is unclear whether this reflects an age-related loss of specialization of cortical brain regions (Park et al., 2004) or a compensatory mechanism (Cabeza et al., 2002). Peelle et al. (2010b) noted that during processing of syntactically complex sentences, older adults engaged middle frontal regions in addition to a “core sentence-processing network” (comprising middle temporal gyrus (MTG) and inferior frontal gyrus (IFG); Peelle et al., 2004; Fiebach et al., 2005) recruited by young adults. The authors interpreted this engagement of additional areas as a compensatory process, whereby the older adults managed to maintain performance despite degeneration of the sensory cortices.

In line with this argumentation, older adults have been hypothesized to engage in more effortful processing during speech comprehension (Pichora-Fuller, 2003). Consistently, Eckert et al. (2008) observed an age-related upregulation of cognitive-control-related frontal areas during an easy word recognition task, while younger adults recruited these areas merely in difficult listening conditions. Harris et al. (2009) further showed that incorrect vs. correct word recognition elicited increased activity in the ACC, but more so in older than younger adults, possibly reflecting an age-related upregulation of error monitoring systems (Sharp et al., 2006). Hence, for solving auditory tasks the reliance on cognitive control appears to increase with age.

It is still largely unknown, however, how older adults adapt to degraded speech. In the current functional MRI study, we were primarily interested in how short-term adaptation to degraded speech and the involvement of cognitive control networks in speech processing changes with age. Young and older listeners heard and repeated back 100 degraded (4-band-vocoded) sentences as well as a control set of interspersed clear-speech sentences. Thus, we could identify age differences in the neural processes related to both, physical degradation of speech (degraded vs. clear sentences) and trial-by-trial fluctuations in comprehension (covariation of BOLD responses with degraded speech comprehension success).

## Materials and methods

### Participants

Sixteen older adults (aged 56–77, mean 67.1 years, 8 female) participated in the study. Their data were analyzed jointly with a cohort of 30 young adults (aged 22–31, mean 25.9 years, 15 female) who had participated in the study reported in Erb et al. (2013). Participants were recruited from the participant database of the Max Planck Institute for Human Cognitive and Brain Sciences according to the following criteria: they were native speakers of German; had no language or neurological disorders; showed dominant right-handedness according to the Edinburgh inventory (Oldfield, 1971) and were naïve to noise-vocoded speech. Younger adults self-reported normal hearing, whereas older adults had normal hearing to moderate hearing loss based on their pure-tone averages which were audiometrically assessed (see below). Participants received financial compensation of € 16, and gave informed consent. Procedures were approved by the local ethics committee (University of Leipzig).

#### Audiometric evaluation

**Figure 1:**
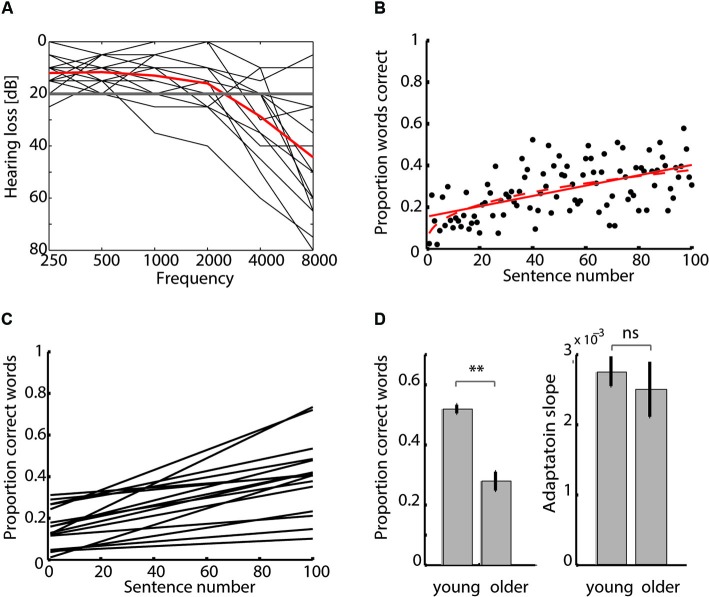
**(A)** Audiograms of older adults. Older participants are affected by varying degrees of sensorineural hearing loss. Hearing loss for each participant’s better ear (black line) and mean over all participants (red line) are shown. **(B)** Mean report scores (averaged over all older participants) with a linear (red solid line) and power law fit (red dashed line). **(C)** Older adults’ adaptation curves. Linear fits to report scores show individual differences in adaptation to degraded speech. The slope of the linear fit was taken as measure for perceptual adaptation to degraded speech (adaptation slope). **(D)** Group comparison of mean speech comprehension and adaptation. Young and older adults differ in mean degraded speech comprehension but not adaptation slopes (**p < 0.001, *ns* = non-significant).

Older adults’ pure-tone thresholds were measured at conventional frequencies from 0.25–8 kHz using an Equinox 2.0 AC-440 audiometer (Interacoustics) in a sound-proof chamber. Older participants’ pure-tone average (PTA; defined as the average thresholds in the listener’s better ear at 1, 2 and 4 kHz) indicated normal hearing (< 25 dB HL) to moderate hearing loss (40–70 dB HL), whereas high-frequency hearing ranged from normal to severe hearing loss (70–95 dB HL; audiograms are shown in Figure 1A). Young participants’ hearing acuity was not tested but all of them self-reported normal hearing.

#### Auditory forward and backward digit span

To measure working memory capacity, all participants were tested with a digit span test, which is part of the revised Wechsler memory scale (WMS-R; Wechsler, 1987). The experimenter read out to the participant lists of single digits between 1 and 9 at a rate of approximately one digit per second. Participants had to immediately repeat the list of digits in the same order (forward digit span) or in the reverse order (backward digit span). The test had seven levels featuring list lengths starting from three digits increasing by one digit up to nine digits for forward digit span, and list lengths from two to eight digits for backward digit span. Each level comprised two items. The participants’ response was marked as correct only if all digits were correctly repeated in the required order. The testing stopped when the participant reported none of the items of a level correctly or when all 14 items had been presented. The level at which the test was terminated was taken as the individual forward or backward digit span measure (Wechsler, 1987).

### Stimuli and experimental design

Stimuli were German SPIN sentences, which control for the predictability of the final word (Kalikow et al., 1977; Erb et al., 2012). Only low-predictability sentences were used in the present study, such that semantic cues were limited and the listener had to rely primarily on acoustic properties of the sentence to understand it. A complete list of these sentences is available in Erb et al. (2012). The sentences were recorded by a female speaker of standard German in a sound-proof chamber. The length of the recorded sentences varied between 1620 and 2760 ms.

Sentences were degraded using 4-band noise vocoding. This procedure divides the speech signal into frequency bands, extracts the amplitude envelope from each band and reapplies it to bandpass-filtered noise carriers. Thus, the spectro-temporal fine structure is smeared while the temporal envelope remains preserved. For envelope extraction, we used a second-order, zero-phase Butterworth lowpass-filter with a cutoff frequency of 400 Hz. Noise-vocoding was applied to all sentences in MATLAB 7.11 as described in Rosen et al. (1999) using four frequency bands spanning 70–9000 Hz that were spaced according to Greenwood’s cochlear frequency-position function (Greenwood, 1990); for the exact cut-off frequencies of the frequency bands see Erb et al. (2012).

Each trial was approximately 9 s long, but actual trial length varied due to cardiac gating (see below). Trials started with a 1 s silent gap, after which participants heard a sentence lasting for approximately 2.5 s. Following stimulus presentation (3.5 s into the trial), a green light (“go”-signal for response) was visually presented and lasted for 3 s. During this time, participants were to respond by repeating the sentence, but were instructed to stop talking when the green light disappeared in order to avoid movement during scan acquisition. After approximately 1 s of silence, scan acquisition with a TR of 2 s was triggered using cardiac gating. Thus, the onset of auditory stimulation preceded the anticipated scan acquisition by approximately 6.5 s. Verbal responses were recorded for later off-line scoring (Eckert et al., 2009; Harris et al., 2009). Responses were scored as proportions of correctly repeated words per sentence (“report scores”, Peelle et al., 2013). Scoring took into account all words of a sentence including function words. The marking scheme was liberal such that errors in declension or conjugation were accepted as correct.

Clear-speech trials were used as a high-level baseline. Clear speech can be assumed to be fully adapted, and therefore to be processed in a stable way over time. This ensured that neural adaptation would not occur in the baseline condition, whereas another type of artificial speech degradation (e.g., rotated speech) might have led to neural adaptation (even in the absence of behavioral adaptation).

Overall, the experiment comprised three conditions, (1) 4-band vocoded sentences (“degraded speech”; 100 trials), (2) clear (non-degraded) sentences (“clear speech”; 24 trials) and (3) trials lacking any auditory stimulation (“silent trials”; 20 trials), summing up to 144 trials in total. Clear speech trials were presented every 5th trial, whereas silent trials were randomly interspersed. Sentences were presented to each participant in one of four different orders; presentation order was counterbalanced across participants.

#### Individual adaptation curves

To measure individual learning rate, we modeled learning curves in two different ways: as power law or as linear performance increase. It has been claimed that learning curves for short-term adaptation to degraded speech are asymptotic such that perceptual learning follows a power law (Peelle et al., 2010a). However, we had previously shown in young adults, that behavioral adaptation to noise-vocoded speech is better described by linear curves than more complex, power-law fits (Erb et al., 2012, 2013).

Here, to test whether in older adults, a linear or a power law function would better describe the data, both functions were fitted to the individual performance scores over time using a least-squares estimation procedure in MATLAB 7.11 (cf. Erb et al., 2012; for an example linear and power law fit to the older group’s average performance see Figure 1B). We compared goodness of fit by determining the Bayesian information criterion (BIC; Schwarz, 1978) of the linear and the power law fit within each subject. BIC values were defined as

(1) $$\text{BIC}=n\times \ln\left(\sigma_{e}^{2}\right)+k\times \ln\left(n\right)$$

where *n* is the number of observations (*n* = 100), $\sigma_{e}^{2}$ is the error variance or sum of squared residuals, *k* is the number of fitted parameters (*k* = 2 for the linear fit, *k* = 3 for the power law fit). Smaller BIC values indicate a better fit (Schwarz, 1978; Priestley, 1981). The BIC increases as a function of $\sigma_{e}^{2}$ and of *k*. Models with higher error variance and more fitted parameters are therefore penalized.

### Experimental procedure

Before participants went into the scanner, they were familiarized with the task by listening to three 8-band vocoded sentences.

To prevent hearing damage due to scanner noise, participants wore Alpine Musicsafe Pro earplugs while in the bore, yielding approximately linear 14-dB reduction in sound pressure up to 8 kHz. Auditory stimuli were delivered through MR-Confon headphones using Presentation software. Presentation level was adjusted for each participant such that loudness was subjectively comfortable and equal across both ears. This ensured that stimuli were presented at a level well above participants’ thresholds in the speech range frequencies such that all participants were able to perceive the sentences. Visual prompts were projected on a screen which participants viewed via a mirror.

### MRI data acquisition

MRI data were collected on a 3-T Siemens Verio scanner. Functional MR images were acquired with a 12-channel head coil using an echo-planar imaging (EPI) sequence [repetition time (TR) ≈ 9000 ms, acquisition time (TA) = 2000 ms, echo time (TE) = 30 ms, flip angle = 90°, 3 mm slice thickness, 30 axial slices (ascending), interslice distance = 1 mm, acquisition matrix of 64 × 64, voxel size = 3 × 3 × 3 mm]. The acquisition matrix was placed such that the *x*-axis was in line with the anterior–posterior commissure (AC–PC). We used a sparse-sampling procedure, where TR was longer than TA, allowing for silent periods to play stimuli and record responses (Hall et al., 1999).

Cardiac gating was applied to avoid movement artifacts caused by the heartbeat in subcortical structures (Von Kriegstein et al., 2008), in which we were especially interested (Erb et al., 2012, 2013). Participants’ heartbeat was monitored using an MR-compatible pulse oximeter (Siemens) attached to their left ring finger. On each trial, after 9 s had elapsed, the scanner waited for the first heartbeat to trigger volume acquisition. Thus, the actual TR was variable.

Following functional imaging, young participants received a T1-weighted structural brain scan with a 32-channel head coil using an MPRAGE sequence [TR = 1300 ms, TE = 3.5 ms, flip angle = 10°, 1 mm slice thickness, 176 sagittal slices, acquisition matrix of 256 × 240, voxel size = 1 mm^3^].

Older adults’ anatomical scans for registration with the functional images were available through the Institute’s brain database. Scanning had been carried out on a 3T Siemens Trio TIM scanner using T1-weighted MPRAGE sequence to acquire 176 sagittal slices, with an acquisition matrix of 256 × 240, yielding a resolution of 1 mm^3^.

For one older participant the scanner had become desynchronized with the presentation script, such that he had to be excluded from the functional MRI data analyses, resulting in a total of 15 older participants included in the analyses. In one young participant, we were only able to acquire 136 (as opposed to 144) scans due to technical problems with cardiac gating.

### Data analysis

Note that all behavioral and MRI analyses of single participants were closely matched to the procedures established previously in the young cohort and published in Erb et al. (2013).

#### Preprocessing

MRI data were analyzed in SPM8 (Wellcome Trust Centre for Neuroimaging, London, UK). Functional images were realigned and unwarped using a fieldmap, coregistered with the structural scan, segmented and normalized to standard space (Montreal Neurological Institute [MNI] space) using the segmentation parameters, and smoothed with an isotropic Gaussian kernel of 8 mm full-width at half-maximum.

#### Statistical analyses

MR images were statistically analyzed in the context of the general linear model (GLM). We set up one model to assess effects speech degradation and effects of trial-by-trial-fluctuations in comprehension. In this model, we included two conditions: degraded and clear speech. To avoid overspecification, silent trials were not modeled in the analyses, but solely used for an initial quality check of the data confirming that sound compared to silent trials yielded large clusters of activity in the auditory cortex. Additionally, a parametric modulator of the degraded speech trials was defined, representing the behavioral report scores. A regressor of no interest, containing report latencies, was added in order to account for differences in speech production (analysis explained in detail below). To assess effects of stimulus clarity, we contrasted degraded against clear speech trials. To reveal effects of trial-by-trial fluctuations in speech comprehension, we assessed correlations with the regressor representing report scores. To look for effects of hearing loss, we correlated PTA on the second level with the contrast degraded > clear speech.

Although the present study was designed to image degraded speech perception, parts of the observed activity may be related to speech production or preparation, because participants overtly repeated back what they had understood starting approximately 3.5 s prior to scan acquisition. In particular, participants’ verbal responses might have been faster for clear relative to degraded speech trials, perhaps leading to partly imaging the BOLD response to speech production, but more so for clear speech trials. Therefore, differences in report latencies might confound the comparison between degraded and clear speech trials. To control for this potential confound, we calculated report latency relative to the onset of the visual response cue. This measure was included at the first level as one single regressor of no interest in the model. For trials where participants did not produce an overt response, the subject-specific mean report latency was entered instead.

All described analyses were whole-brain analyses. Regressors were modeled using a finite impulse response (FIR) comprising one bin. A high-pass filter with a cutoff of 1024 s was applied to eliminate low-frequency noise. No correction for serial autocorrelation was necessary because of the long TR in the sparse-sampling acquisition.

Second level statistics were calculated using a one-sample *t-*test and group differences were assessed using a two-samples *t*-test. We recognize that comparisons between groups of different sample sizes (here: 15 older adults vs. 30 younger adults) are problematic; especially when variance differs between groups, the group with the larger variance should comprise more samples (Samanez-Larkin and D’Esposito, 2008). There is evidence, however, that BOLD signal variability actually decreases in older adults (Garrett et al., 2010). Further, we wanted to avoid discarding data which were already available for the 30 young adults, resulting in a larger sample size of the young compared to the older adults.

We are aware of the problem that hemodynamics likely change with age (e.g., due to vascular changes), such that simple group differences in the BOLD signal could possibly reflect differences in neurovascular coupling rather than actual differences in neural processing. To overcome this issue, we only tested for age group × condition interactions when assessing age effects on neural processing (Samanez-Larkin and D’Esposito, 2008).

Group inferences are reported at a threshold of *p* < 0.001 and a cluster-extent of *k* > 20 to correct for inflated type I error at the whole-brain level, as based on a Monte Carlo Simulation (Slotnick et al., 2003). *T*-statistic maps were transformed to *Z*-statistic maps using spm_t2z.m, and overlaid and displayed on the ch2 template in MNI space included with MRIcron (Rorden and Brett, 2000).

#### Regions of interest analyses

In order to extract measures of percent signal change in the regions identified by the whole-brain analyses described above, we defined regions of interest (ROIs) using the SPM toolbox MarsBar (Brett et al., 2002). ROIs were defined as spheres with a radius of 3 mm centered on the identified peak coordinates. Voxels within an ROI were aggregated into a single contrast estimate using the first eigenvariate.

## Results

### Behavioral results

#### Vocoded speech comprehension

Older adults reported on average 28.0 ± 2.8 (mean ± SEM) % words correctly per degraded sentence. Performance in clear trials was at 98.0 ± 1.4 % correct. In comparison, young adults’ degraded speech recognition was substantially better (*t*(44) = 8.23, *p* < 0.001), with on average 51.9 ± 1.4 % words correct per degraded sentence and 99.7 ± 0.2 % correct per clear sentence (Figure 1D, left).

#### Perceptual adaptation

We compared whether a linear or power-law fit would better describe the report scores’ increase over time by calculating BIC for each fit and each older participant (Figures 1B, C). The BIC scores for the linear fits (median 222.09, range 95.21–268.48) were smaller than those for power law fits (median 226.69, range 99.81–273.09), as shown by a Wilcoxon signed-rank test (*p* < 0.001), indicating that the linear curve better fit the behavioral data. In the young participants, we had also shown that linear fits were more adequate than the power law fits to describe participants’ learning curves (see Erb et al., 2013). Thus, the slope of the linear fit (adaptation slope) was taken as a measure of individual perceptual adaptation to degraded speech.

The BIC compares goodness of fit between different models but does not give an estimate of absolute goodness of fit. Absolute goodness of fit as measured by *R*^2^ in the older adults amounted to *R*^2^ = 0.061 ± 0.01 (mean ± SEM) for the power law fit and *R*^2^ = 0.072 ± 0.016 for the linear fit. *R*^2^ did not differ between the linear and the power law fit, as shown by a Mann-Whitney *U*-test (*p* = 0.95). Note however, that a direct comparison of *R*^2^ between different models does not allow for a fair comparison, as *R*^2^ does not take into account the number of fitted parameters.

In order to test whether goodness of fit differed between age groups, we compared *R*^2^ of the linear fits in the two groups. *R*^2^ in the older adults did not differ from *R*^2^ in the younger adults (0.065 ± 0.009), as shown by a Mann-Whitney *U-*test (*p* = 0.92), indicating that the goodness of fit was comparable in older and younger adults. Although these single-subject *R*^2^ values are relatively small (only approximately 7% of the variance is explained by the fitted model), when averaging over the 16 older adults, mean report scores correlated highly with sentence number (*R*^2^ = 0.32, *p* < 0.001). Davis et al. (2005) have reported similar correlation coefficients for mean report scores with sentence number in their vocoded speech learning study (but did not report single-subject *R*^2^-values).

To confirm that the slopes of the linear fits were a sensible measure of learning, we used another more traditional measure of learning. For each participant, we subtracted the mean performance over the last 20 trials from the mean performance over the first 20 trials (Bent et al., 2009; Eisner et al., 2010). This performance difference (Δ performance) between the beginning and end of the experiment amounted to 0.22 ± 0.03 proportion correct (mean ± SEM) in the older adults and 0.27 ± 0.02 proportion correct in the younger adults. Across age groups, Δ performance was highly correlated with the adaptation slopes (*r* = 0.91; *p* < 0.001).

To test whether older and younger adults differed in their rate of learning, we compared both the Δ performance and adaptation slopes between groups. According to a two-samples *t*-test, there was neither a difference in Δ performance between older and young adults (*t*(44) = −1.25, *p* = 0.22), nor in the adaptation slopes of older (2.5 ± 0.39 × 10^−3^, mean ± SEM) and young adults (2.7 ± 0.21 × 10^−3^; *t*(44) = −0.65, *p* = 0.52), indicating that both groups were comparable in the rate with which they adapted to degraded speech (Figure 1D, right).

Finally, to exclude the possibility that variability in the adaptation curves (shown in Figure 1C) was a consequence of the counterbalancing of material across participants (in four different presentation orders), we tested whether presentation order had an effect on adaptation slope. A Kruskal-Wallis test was not significant (*χ*^2^(15) = 4.34, *p* = 0.23), indicating that the fact that different listeners received different materials at different time points did not influence adaptation.

#### Spearman’s correlations

In order to examine whether in older adults, adaptation slope was related to other factors (i.e., age, forward and backward digit span, PTA, mean performance), a two-tailed Spearman’s correlation was calculated for all pairs of variables. We adjusted for multiple comparisons by controlling the false discovery rate (Benjamini and Hochberg, 1995), which resulted in a critical *p* = 0.019 at a false discovery rate of *q* = 0.05 (“significant”) and *p* = 0.039 at *q* = 0.1 (“non-significant trend”).

**Figure 2:**
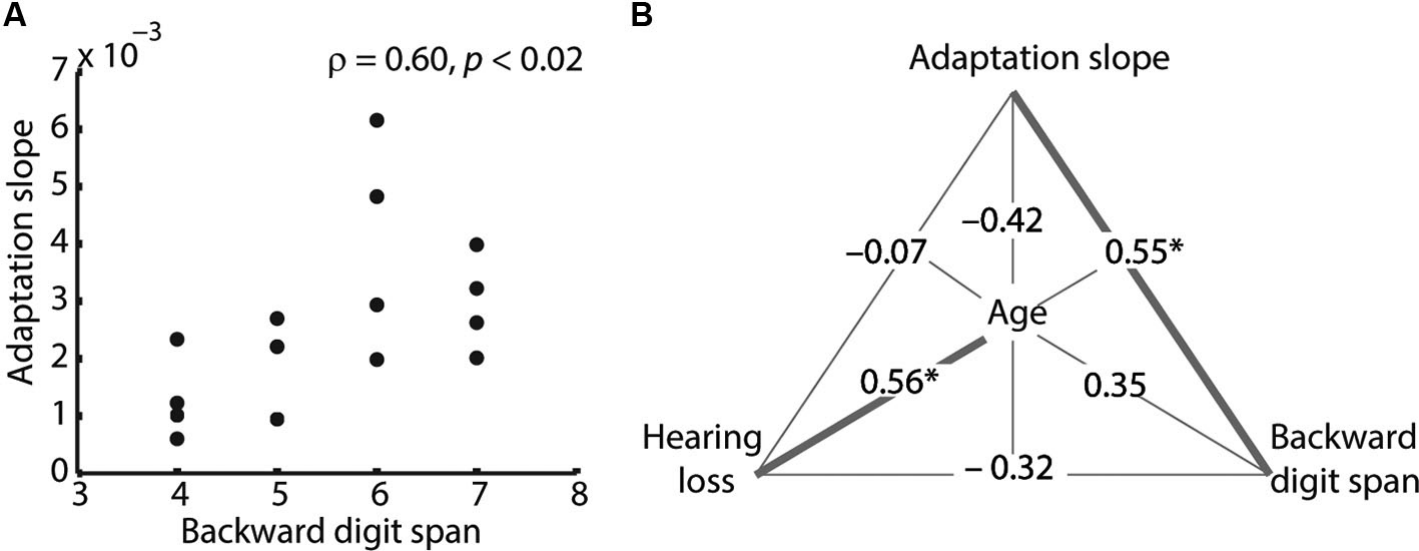
**Spearman’s correlations between behavioral measures. (A)** Adaptation slope correlated positively with backward digit span. This correlation remained significant, when the outlier participant with a slope of 0.006 was removed (*ρ* = 0.62, *p* = 0.18). **(B)** Partial correlations between adaptation slope, age, backward digit span and hearing loss (pure-tone average; PTA). Spearman’s correlation coefficients are shown. Note that two measures are partialled out at a time; for example, the correlation between adaptation slope and digit span of *ρ* = 0.55 is controlled for age and hearing loss. Only the adaptation–digit span correlation and the age–hearing loss correlation remain significant in the partial correlation. Significant correlations are shown as bold lines (* *p* < 0.05).

Within the older adults, adaptation slope correlated positively with backward digit span (Figure 2A and Table 1), indicating that listeners with a larger working memory capacity adapted faster to degraded speech. Note that this correlation remained significant, when the outlier participant with a slope of 0.006 was removed (*ρ* = 0.62, *p* = 0.18). PTA and age showed a non-significant trend for a negative correlation with adaptation slope, meaning that older age and hearing loss were associated with slower adaptation rates. Similarly, older age and PTA were negatively related to average speech comprehension performance. Finally, age also correlated significantly with PTA, indicating that older adults had greater hearing loss (Table 1).

**Table 1:** **Spearman’s correlations between behavioral measures**.

	**Slope**	**Age**	**DSF**	**DSB**	**PTA**	**Perform**
**Slope**		−0.54+	0.37	0.60*	−0.55+	0.45
**Age**			0.01	−0.18	0.65*	−0.67*
**DSF**				0.25	−0.26	0.10
**DSB**					−0.44	0.09
**PTA**						−0.61*
**Perform**						

Significant correlations are shown in bold: * significant at *q* < 0.05; + significant at *q* < 0.1; Slope: adaptation slope, DSF: digit span forward, DSB: digit span backward, PTA: pure-tone average, Perform: Mean vocoded speech comprehension.

To analyze more closely the relationship between adaptation slope, age, digit span and hearing loss, we calculated Spearman’s partial correlation coefficients between these four variables of interest, with two variables partialled out at a time (Figure 2B). Adaptation slope still correlated significantly with backward digit span, even after partialling out age and hearing loss, indicating that the latter could not explain the relationship between working memory and adaptation. The correlation between PTA and age also remained significant in the partial correlation. On the other hand, the non-significant trend for a negative correlation of adaptation slope with hearing loss and age broke down in the partial correlation (Figure 2B).

### Functional MRI results in older adults

The results reported below refer to the group of older adults exclusively. For the cohort of young adults, activation clusters and coordinates of peak activity are described in detail in Erb et al. (2013).

#### Degraded speech processing

**Figure 3:**
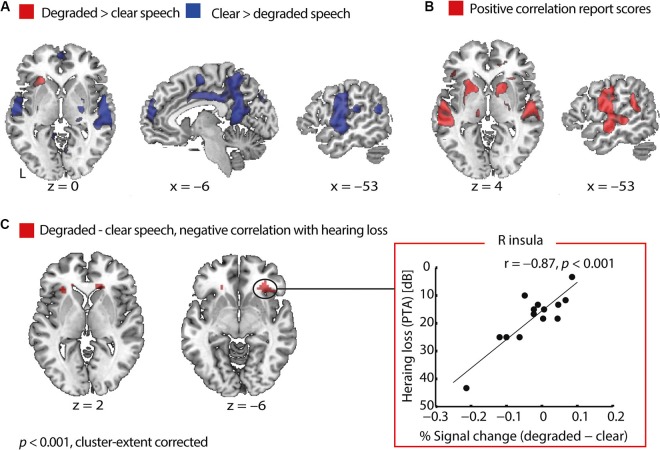
**Functional MRI results in older adults. (A)** Areas sensitive to speech degradation. In older adults, degraded relative to clear speech perception activated the anterior insula, whereas clear more than degraded speech activated a network comprising the precentral gyrus, the temporal cortices, supramarginal gyrus, right putamen, posterior cingulate cortex and angular gyrus bilaterally. **(B)** Areas varying with speech comprehension. With better speech comprehension on a given trial, activity increased in a network comprising the auditory cortices, precentral gyrus, left angular gyrus, putamen and middle frontal gyrus. There were no negative correlations with trial-by-trial report scores. **(C)** Negative correlation of hearing loss with activity related to degraded speech processing. Pure-tone average (PTA) correlated negatively with the degraded > clear speech contrast in the right and left anterior insula. The plot of this correlation (right panel) shows that older adults with better hearing acuity had an increased BOLD signal for degraded compared to clear speech in the right anterior insula. Conversely, listeners affected by more severe hearing loss had elevated activity for clear relative to degraded speech. The correlation remains significant when removing the outlier participant with a PTA of 43 dB (*r* = −0.76, *p* = 0.002) and when partialling out age (*r* = −0.79, *p* = 0.001). Note that the y-axis is flipped such that better hearing acuity is plotted higher up.

To reveal regions that are engaged in degraded speech processing, we compared degraded with clear speech trials. At a cluster-extent corrected threshold of *p* < 0.001, degraded compared to clear speech elicited an increased BOLD signal in the left anterior insula. On the other hand, clear compared to degraded speech yielded higher activations bilaterally in the precentral gyrus spanning the temporal cortices, supramarginal gyrus (SMG), right putamen, posterior cingulate cortex and angular gyrus bilaterally (Figure 3A).

#### Trial-by-trial fluctuations in degraded speech comprehension

To identify areas where the BOLD signal varied with trial-by-trial fluctuations in speech comprehension, we tested for correlations with the behavioral report scores. The hemodynamic response linearly increased with comprehension of degraded speech in bilateral temporal cortices comprising Heschl’s gyrus, the middle temporal gyrus, the precentral gyrus bilaterally, the putamen bilaterally, left angular gyrus, and middle frontal gyrus (Figure 3B). Although scans might have been sensitive to both speech perception and production, we controlled for report latency (see Section Materials and Methods) to model the hemodynamic response to auditory input rather than speech production. Note, however, that including a regressor for report latency might not completely control for production-related activity, such that motor cortical activity (apparent in Figures 3A, B), could be due to more speech production during more intelligible compared to less intelligible trials.

#### Correlation with hearing loss

We further tested, whether hearing acuity had an influence on neural processing. For the contrast degraded > clear speech, we found a negative correlation with PTA in the right and left anterior insula (Table 2 and Figure 3C). The correlation showed the following pattern: Older adults with better hearing acuity had elevated activity for degraded compared to clear speech. Conversely, listeners with greater hearing loss had an increased BOLD signal for clear relative to degraded speech in the anterior insula (Figure 3C, right panel). This Pearson’s correlation between insula dynamics and hearing loss was significant even after partialling out age (*r* = −0.79, *p* = 0.001), confirming that the correlation was not driven by age.

**Table 2:** **Overview of MRI activation clusters showing a correlation with hearing loss or significant group × condition interactions, thresholded at *p* < 0.001 (cluster-extent corrected)**.

**Location**	**MNI-Coordinates**			***Z*-Score**	**Extent (mm^3^)**
**Degraded > clear speech, negative correlation with PTA (older adults)**					
R anterior insula	33	35	−5	4.38	432
L anterior insula	−30	26	1	4.08	207
**Degraded > clear speech, young > older**					
L SMA/ACC	−3	26	40	3.58	207
**Positive correlation with trial-by-trial comprehension fluctuations, older > young**					
R MFG	33	35	34	4.27	754
**Positive correlation with trial-by-trial comprehension fluctuations, young > older**					
L fusiform gyrus	−30	−43	−11	4.65	855
R cerebellum	12	−49	−35	3.94	477
Posterior cingulate gyrus	0	−58	28	4.49	648

*L: left, R: right, ACC: anterior cingulate cortex, MFG: middle frontal gyrus, SMA: supplementary motor area*.

### Functional MRI differences between young and older adults

#### Degraded speech processing

**Figure 4:**
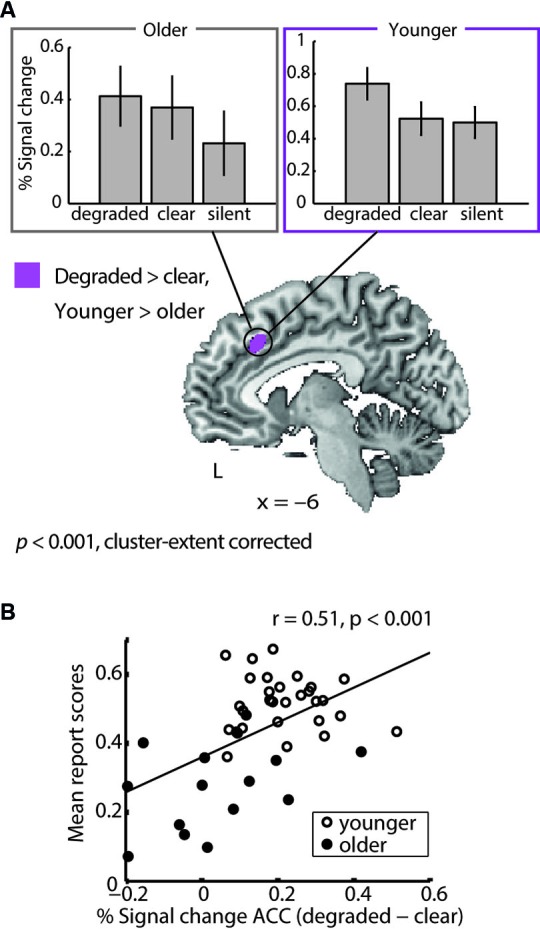
**(A)** Age group × degradation interaction.**** Young adults showed a greater difference in anterior cingulate cortex (ACC) activity between degraded and clear speech than older adults. This age group × degradation interaction was driven by a reduced dynamic range in older adults, who displayed increased levels of ACC activation during perception of both clear and degraded speech. This is apparent in the bar graphs showing % signal change for both groups and conditions (upper panel). For comparison, signal change for silent trials is shown, illustrating that older but not younger adults upregulate ACC activity for clear compared to silent trials. **(B)** Correlation of ACC dynamics with performance. A post-hoc ROI analysis showed that across groups, individuals with a better overall degraded speech comprehension (mean report scores) showed a higher differentiation (degraded minus clear speech) in ACC activity.

Generally, older and younger adults showed largely overlapping activations during degraded speech processing (see Erb et al., 2013 for the activation clusters in young adults). However, the ACC exhibited a group difference: young adults showed a higher increase in ACC activity when comparing degraded to clear speech than older adults did. This age group × degradation interaction was driven by a reduced dynamic range in older adults, who displayed persistently elevated levels of ACC activity in both conditions (Figure 4A and Table 2).

Extracting individual percentage signal change values from the ACC region of interest (as identified by the age group × degradation interaction) showed that, across groups, activity dynamics in the ACC were related to performance: Individuals with an overall better degraded speech comprehension showed a higher ACC differentiation for degraded vs. clear speech (Figure 4B). However, this correlation was driven by age, because the correlation was not significant in both groups separately (Pearson’s correlation in young adults: *r* = −0.07, *p* = 0.73; older adults: *r* = 0.39, *p* = 0.16) and the correlation broke down when controlling for age (*r* = 0.15, *p* = 0.34). Within older adults, however, the correlation reached a non-significant trend, when partialling out age (*r* = 0.46, *p* = 0.09). However, the age group × mean performance interaction in the ACC BOLD signal failed to reach significance (*t*(41) = 1.5, *p* = 0.14).

#### Trial-by-trial fluctuations in degraded speech comprehension

**Figure 5:**
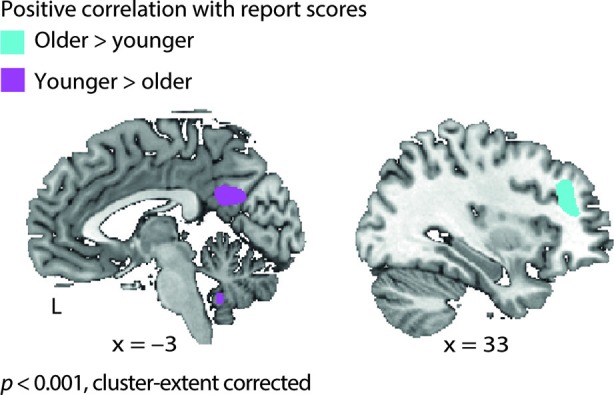
**Age group × comprehension interaction**. In older adults the hemodynamic response in middle frontal gyrus covaried with comprehension of degraded speech on a trial-by-trial basis (i.e., report scores); young adults did not exhibit such a correlation. Incontrast, young adults showed additional correlations with report scores in the posterior cingulate cortex, left fusiform gyrus and right cerebellum (see Table 2).

Following up on the comprehension-dependent fluctuations observed in the cohort of young listeners (Erb et al., 2013), we also tested for such fluctuations in the older adults. While observing again substantial overlap between groups, an age group × comprehension interaction, also in prefrontal cortex, was manifest: Older adults’ BOLD signal positively correlated with comprehension (i.e., report scores) in the middle frontal gyrus (MFG). Young adults did not show such a correlation in MFG. Young adults, on the other hand, exhibited additional correlations with comprehension in the left fusiform gyrus, right cerebellum and posterior cingulate cortex, where older adults did not show a correlation (Figure 5 and Table 2).

## Discussion

The current study intended to compare degraded speech processing between young and older adults to characterize how underlying neural mechanisms change with age. The main results can be summarized as follows: Although degraded speech comprehension overall appeared deteriorated in older adults, both older and younger adults adapted to degraded speech at the same rate. Within older listeners, better working memory predicted faster adaptation rates and hearing loss predicted worse speech comprehension. Hearing loss was related to a distinct activation pattern in the anterior insula during degraded speech processing. Young listeners showed an expected modulation of ACC activity depending on task difficulty (i.e., degraded greater than clear speech), whereas older adults displayed elevated levels of ACC activity throughout, consistent with a persistent upregulation in this cognitive-control related area. Within the ACC, a greater dynamic range predicted better speech comprehension. Finally, for correctly comprehended degraded speech trials, older listeners recruited middle frontal regions in addition to a core speech comprehension network which younger listeners relied on, most likely reflecting a compensatory mechanism. We will now discuss these results in more detail.

### Perceptual adaptation is comparable in young and older adults

Even though degraded speech comprehension was substantially reduced in older listeners, their ability to gradually adapt to degraded speech over the course of the experiment was preserved, as the slopes of their linear fits (adaptation curves) did not differ from young adults (Figure 1C). Yet, as the *R*^2^-values were relatively low (but cf. Davis et al., 2005, for comparable *R*^2^-values), it remains questionable what no difference between age groups in a parameter of a badly fitting model actually means. Importantly however, we have shown that a second, model-free measure of learning (Δ performance) is strongly correlated with adaptation slope, and does not differ between age groups either. We take this as evidence that adaptation is comparable in young and older adults.

This result is consistent with a finding by Peelle and Wingfield (2005) who had shown that older adults’ (aged 65–78) perceptual learning of both time-compressed and spectrally shifted noise-vocoded speech was comparable to those of young adults: when older adults’ starting accuracy was equated with young adults, both groups’ speech comprehension improved at the same rate when listening to 20 degraded sentences. Thus, older and younger adults appear to show equivalent behavioral adaptation to degraded speech. However, this does not warrant that the underlying neural mechanisms are identical.

Older listeners with a better short-term memory adapted faster to degraded speech. The correlation of adaptation with backward but not forward digit span reached significance. The latter demands simple maintenance and repetition. In contrast, backward digit span requires the listener to perform an operation on the items held in the memory buffer (i.e., inverse the order of the digits) and has specific demands on central executive mechanisms (Baddeley, 2012), which are supposedly involved in effortful speech perception. Rabbitt (1968) suggested an effortful hypothesis, according to which working memory becomes a limiting factor when perceptual effort is required in degraded speech comprehension: Masked words disrupt the short-term memory buffer, because resources that would otherwise be available for encoding in short-term memory are diverted for perceptual effort (Piquado et al., 2010). Our result lends further support to the hypothesis that such cognitive and perceptual abilities become coupled more tightly at an older age (Baltes and Lindenberger, 1997).

### Hearing loss deteriorates degraded speech comprehension and alters insula dynamics

One aim here was to identify how individuals with hearing loss recognize and process degraded speech. Unsurprisingly, a decline in hearing acuity was associated with worse average degraded speech comprehension in older adults. This is best explained by the fact that on top of the exogenous signal degradation (i.e., 4-band vocoding), hearing loss endogenously distorts the signal and reduces audibility (Pichora-Fuller and Souza, 2003).

In addition, hearing loss was accompanied by changes in central neural mechanisms. While older adults with worse hearing activated the anterior insula more for clear than degraded speech, better-hearing older listeners had increased anterior insula activity during degraded relative to clear speech perception. Importantly, this activation pattern in the insula could not be explained by age. In Erb et al. (2013), we had shown that younger adults rely on the anterior insula in difficult listening (see Erb et al., 2013). Here, it appears that only better-hearing older adults succeed to recruit the anterior insula in adverse listening conditions, whereas older adults affected by substantial hearing loss show the inverse dynamics. These hearing loss effects were found even though speech was presented at an audible level for each participant, supporting the notion that these are centrally-driven changes.

The insula together with the ACC have commonly been termed the “cingulo-opercular” system (e.g., Harris et al., 2009). Here, an interesting dissociation between insula and ACC dynamics emerges: Anterior insula activation was affected by hearing loss (independent of age), while ACC activity was altered by age (see below).

There is accumulating evidence that the insula plays a crucial role in top-down, executive processes (Eckert et al., 2009; Menon and Uddin, 2010; Sterzer and Kleinschmidt, 2010; Adank, 2012). For example, Wild et al. (2012) showed that in young listeners, the insula exhibited an enhanced BOLD signal when listeners attended to speech (rather than a distracter), and the speech signal was degraded (vocoded rather than clear). Activation in the anterior insula further scaled with task-difficulty of a temporal non-speech auditory task, dependent on attention (Henry et al., 2013).

The current results are somewhat more complicated, as insula engagement depended on the extent of hearing loss; only those listeners with very mild, age-typical hearing loss (< 20 dB HL) exhibit such a pattern, while more hearing-impaired listeners did recruit insular cortex into clear-speech (i.e., already at milder task demands), but less so into degraded-speech processing (higher task demands). Intriguingly, this pattern of insula activation is reminiscent of the Crunch-hypothesis, according to which older adults show a load-dependent inverse *u*-shaped pattern of activity in cognitive-control-related areas (Reuter-Lorenz and Cappell, 2008). While this finding deserves thorough follow-up experimentation, the insula seems to be crucial for cognitive control (Eckert et al., 2009) and the observed alteration of its hemodynamics might add up to declines of the peripheral auditory system and manifest in the observed deterioration in speech recognition.

### Persistent upregulation of anterior cingulate activity reflects increased cognitive control in older adults

The main objective of the current study was to identify age differences in the neural systems supporting degraded speech processing. We found an age group × degradation interaction in the ACC, where young listeners showed a higher activation difference between degraded and clear speech than older adults. The latter displayed elevated levels of ACC activation during both conditions, indicating a reduced dynamic range in older adults. Thus, the older adults’ BOLD signal in the ACC appears to be less informative and flexible, as it is differentiating less between a degraded and clear speech input. This is consistent with an age-related decrease in the variability of the hemodynamic signal (Garrett et al., 2010).

As we intended to compare the results in the older group to the ones obtained in young adults (Erb et al., 2013), we normalized older adults to a young adult (MNI) template. This can be problematic due to group differences in brain morphology. For example, partial volume effects, that is, sampling both grey and white matter in one voxel, may increase in older adults, who commonly exhibit gray matter atrophy in frontal regions. One solution to overcome this problem is to only test for the interaction (Samanez-Larkin and D’Esposito, 2008). As we found an age group × condition interaction in the ACC, it is unlikely that older adults’ smaller dynamic range in ACC activity could be driven by the non-diffeomorphic normalization of older adults to a young adult (MNI) template.

Age-related functional and structural changes in frontal lobe systems supporting cognitive control have been previously noted (Cabeza et al., 2004; Colcombe et al., 2005; Sharp et al., 2006; Eckert et al., 2008; Harris et al., 2009). For example, Harris et al. (2009) showed that ACC activity increases for incorrect compared to correct word recognition, but more so in older adults. The authors linked this to auditory cortex architecture, showing that ACC recruitment correlates with age-related neurodegeneration of the auditory cortex. Along the same line, Sharp et al. (2006) showed that aging is accompanied by greater cognitive control, as indexed by higher ACC and prefrontal cortex activity during semantic and syllabic decisions on noise-vocoded words. Both Sharp et al. (2006) and Harris et al. (2009) observed that activity in ACC increased with age and was detrimental to performance. Therefore, they interpreted the age-related increase of ACC activation as upregulation of error monitoring systems (Dosenbach et al., 2006, 2007).

In contrast, the present findings show that a higher dynamic range of ACC activity (i.e., the degree to which the ACC became relatively engaged and disengaged in degraded and clear speech, respectively) was associated with better speech comprehension. However, this correlation was best explained by age, as the correlation broke down when correlating groups separately. The following picture emerges: Dynamic range of ACC activity decreases with age which in turn is detrimental to speech comprehension. Rather than playing a compensatory role for deficits due to aging (Cabeza et al., 2002), the observed ACC dynamics might reflect a generalized upregulation of cognitive control with age irrespective of task difficulty (see also the discussion of dedifferentiation vs. compensation hypothesis below).

### Compensatory prefrontal activity during speech comprehension in older adults

For successful speech comprehension, younger adults’ activated a perisylvian network (Erb et al., 2013), where the BOLD signal was tightly coupled to actual speech comprehension (i.e., behavioral report scores), rather than acoustic properties of the sentences. Older adults additionally showed a correlation with report scores in middle frontal gyrus. Eckert et al. (2008) similarly demonstrated that older adults engage the MFG when words are most intelligible. However, their design varied speech intelligibility by low-pass filtering words such that effects due to acoustic differences could not be disentangled from actual comprehension. In contrast, the current study held physical stimulus properties constant (i.e., 4-band vocoding) and thus identified regions where activation varied with actual speech comprehension (i.e., behavioral report scores). Therefore, the present data provide evidence that additional MFG activation observed in older adults is related to comprehension rather than physical speech clarity.

Age-related additional recruitment of middle frontal or lateral prefrontal cortex has been repeatedly observed, for example, during working memory tasks (Cabeza et al., 2002), visual attention (Cabeza et al., 2004), word recognition (Eckert et al., 2008), and for processing of syntactically complex sentences (Peelle et al., 2010b). Two hypotheses have been put forward to explain the frequently observed recruitment of additional brain regions by older adults not observed in young adults: The dedifferentiation hypothesis (Baltes and Lindenberger, 1997) interprets the extra activation as difficulties in recruiting specialized neural mechanisms for the relevant task, hence as a loss of neural specialization (Park et al., 2004). Such a generalized non-functional spread of activation has been attributed to a deficit in neurotransmission with a decrease in signal-to-noise ratio in neural firing (Li and Lindenberger, 1999). If this hypothesis is true, engagement of additional regions should not correlate with task performance. On the other hand, the compensation hypothesis suggests that recruitment of additional brain regions plays a compensatory role, for example in counteracting performance decline due to neurodegeneration in specialized brain areas (e.g., the auditory cortex; Harris et al., 2009; Peelle et al., 2011; Eckert et al., 2012), and should therefore improve performance (Cabeza et al., 2002; Heuninckx et al., 2008).

In the current study, engagement of the MFG in older adults covaried with report scores, that is, MFG activity did in fact increase with better performance. We take this as evidence for a compensatory mechanism in older adults, whereby the MFG is recruited in addition to the core speech comprehension network (Erb et al., 2013) when speech comprehension succeeds. In sum, our data contribute to, but cannot solve, the ongoing debate of dedifferentiation vs. compensation, in that the age-group-differences observed in two prefrontal areas, ACC and MFG, are to be interpreted with opposing conclusions regarding the compensation hypothesis by Cabeza et al. (2002, 2004).

## Conclusions

Our results show distinct age-related changes of responses in prefrontal cortex. Higher anterior cingulate and middle frontal gyrus activities are found associated with better performance in adverse listening conditions. However, unlike younger adults, older adults do not succeed in selectively modulating ACC activity depending on listening difficulty, but exhibit generalized upregulated levels of ACC activity also in easy listening conditions. In contrast, MFG activity appears to be truly compensatory, as older adults recruit frontal areas in addition to a speech comprehension network when comprehension succeeds. Moreover, more hearing-impaired older adults involve the anterior insula more even in clear speech comprehension. As all three structures have been linked to cognitive control, the results provide further evidence that older adults increasingly rely on cognitive control networks when adapting to challenging listening conditions, at the potential expense of these systems’ dynamic range.

## Author contributions

Julia Erb designed and conducted research, analyzed the data and wrote the paper and Jonas Obleser designed research, analyzed the data and wrote the paper.

## Conflict of interest statement

The authors declare that the research was conducted in the absence of any commercial or financial relationships that could be construed as a potential conflict of interest.
